# Plasticity and antigen presentation by group 3 innate lymphoid cells in colorectal cancer

**DOI:** 10.3389/fimmu.2026.1783909

**Published:** 2026-06-09

**Authors:** Lu Qiao, Jingwen Zhang, Jiaxi Li, Xia Li, Xiaoxia He, Mingyu Zhang, Jingkun Lu, Xuan Zhang, Jing Dong, Gesi Tao, Yue Wang, Jiaxian Cui, Lili Bao, Pengwei Zhao

**Affiliations:** 1 Laboratory of Microbiology and Immunology, School of Basic Medical Science, Inner Mongolia Medical University, Hohhot, China; 2 Department of Clinical Laboratory, Affiliated Hospital of Inner Mongolia Medical University, Hohhot, China

**Keywords:** antigen presentation, colorectal cancer, group 3 innate lymphoid cells, human beta-defensin 1, microbiota

## Abstract

Colorectal cancer (CRC) develops within a mucosal ecosystem where the microbiota, epithelial barrier, and group 3 innate lymphoid cells (ILC3) jointly shape immune tone. ILC3 plasticity spans both tissue-protective and inflammatory states, and MHC II-dependent antigen presentation-like activity of ILC3 is associated with microbiota-directed CD4^+^ T cell and IgA responses. In CRC, chronic inflammation and dysbiosis drive a shift toward barrier-damaging ILC3 programs, attenuate epithelial antimicrobial defenses, and defocus microbiota-directed immunity. These alterations correlate with more aggressive tumor behavior and poorer responses to immune checkpoint therapy. We integrate this evidence into a feed-forward model in which impaired crosstalk among ILC3, the epithelium, and the microbiota promotes barrier weakening, inflammatory amplification, and tumor progression. Within this framework, we propose biomarker panels that capture ILC3 state, barrier integrity, and DEFB1 expression, together with therapeutic strategies targeting cytokine pathways, metabolism, microbiota structure, and immune checkpoints.

## 1 Introduction

In the past decade, colorectal cancer (CRC) has undergone a notable epidemiological shift: in several countries, overall incidence has stabilized or declined, yet the proportion of advanced-stage diagnoses is increasing and new cases are occurring more often in individuals under 50 years of age (1). These patterns support viewing CRC as a chronic pathological process within a highly active intestinal mucosal ecosystem rather than solely as a mutation-driven malignancy of older adults (2, 3), and argue for moving beyond a tumor cell–centered, T cell–focused paradigm to include innate networks at the mucosal interface (4–7).

Group 3 innate lymphoid cells (ILC3) are tissue-resident lymphocytes enriched in the intestinal lamina propria and secondary lymphoid structures. They express RORγt and rapidly produce effector cytokines such as interleukin (IL)-22 and IL-17 in response to integrated cues from commensals, epithelium, and stroma, thereby coordinating barrier repair, antimicrobial peptide production, and tolerance to luminal antigens (8). ILC3 thus position themselves at the intersection of tissue homeostasis, microbiota, and immunity. In tumors, they display context-dependent functions: on one hand, they support antitumor immunity by recruiting and activating local immune cells; on the other hand, under IL-23-dominated conditions, they may promote angiogenesis, stromal remodeling, and the accumulation of immunosuppressive cells (9). Lineage plasticity further increases their functional heterogeneity. Specifically, IL-12 and TGF-β drive ILC3 toward ILC1-like or NK-like features, with increased interferon-gamma (IFN-γ) production and acquisition of cytotoxic potential, changes that can be linked either to enhanced antitumor activity or to persistent chronic inflammation (9).

A major route by which ILC3 shape adaptive immunity is an MHC II-dependent antigen presentation-like program. In murine colon cancer models, MHC II loss in ILC3 is associated with reduced ILC3 abundance and increased plasticity, disrupting the balance between ILC3 and T cells, promoting invasive growth, and leading to resistance to PD-1 blockade. By contrast, preserved MHC II expression supports more effective type 1 immunity against microbiota-associated antigens (10). These opposing outcomes underscore that ILC3 do not act through a fixed program but rather integrate signals from the local microenvironment to modulate adaptive immunity, helping to shape microbiota-specific CD4^+^ T cell responses and immunoglobulin A (IgA) targeting rather than acting solely as upstream cytokine producers.

In this review, we integrate these observations into a tri-directional framework that organizes the subsequent sections. The first axis centers on ILC3 plasticity, linking IL-22, IL-17, and IFN-γ outputs to epithelial repair, microbial control, and T cell polarization. The second axis is defined by MHC II-dependent antigen presentation-like functions of ILC3, which translate recognition of commensal and carcinogenic microbes into shifts in Th17/Treg balance and IgA specificity. The third axis is the epithelium–microbiota–hBD-1 barrier system, which continuously sculpts microbiota composition and mucosal inflammatory thresholds at the level of both physical and chemical barriers. Within this framework, CRC emerges as a syndrome of multiple interlocking loops drifting out of balance. After detailing these three axes, we assemble them into a feed-forward model of tri-directional crosstalk, propose measurable biomarker panels and therapeutic strategies, and conclude with a summary of key principles and unresolved questions.

Before developing this framework, however, it is important to note that ILC3 plasticity and heterogeneity in CRC are highly context dependent. Cytokine milieu, microbial composition, and stromal signals vary across patient cohorts, and these variations correlate with different distributions of ILC3 and ILC1-like populations and with distinct functional states (11). Within this broader view, ILC3 have also been discussed in terms of trained innate immunity, whereby inflammatory or microbial stimuli imprint durable epigenetic and metabolic programs on tissue-resident cells and generate “memory-like” ILC3 with biased response profiles (12). Single-cell analyses of human colon further support this heterogeneity: in normal tissue, ILC1, ILC3, and mixed ILC3/NK populations dominate, whereas CRC lesions contain additional ILC1-like and ILC2 subsets; NKp44^+^ ILC3 are associated with tertiary lymphoid structures in early-stage disease and decline in advanced T3/T4 tumors in parallel with reduced expression of chemokines such as CXCL13, CCL19, and CCL21 (13). ILC3 have been described as integrators of environmental cues that maintain commensal tolerance, pathogen control, and epithelial repair; disruption of these programs is associated with infection, inflammatory bowel disease, and tumorigenesis (14). In CRC, this means that the ILC3 compartment reflects both the current tumor microenvironment and the history of epithelial injury, microbial shifts, and prior inflammation, providing a plausible explanation for discordant ILC3-related findings across studies and underscoring their potential as biomarkers and therapeutic targets at the mucosal interface (15).

## 2 ILC3 plasticity in colorectal cancer

ILC3 functions in colorectal cancer were shaped by their ability to move along a type 3 to type 1 spectrum. In this section, key environmental cues that biased this plasticity were summarized, and regulatory nodes that constrained lineage drift were highlighted. Emphasis was placed on signals that were likely to operate at the mucosal interface, including cytokines, metabolites, and tissue rhythms.

### 2.1 Cytokine and metabolic cues

In inflammatory and tumor settings, cytokine combinations—including IL-1β, IL-23, IL-12, TGF-β, IL-15, and IL-18—shaped ILC3 fate. IL-23 together with IL-1β favored a barrier-protective program dominated by IL-22 and IL-17. IL-12 together with TGF-β promoted T-bet upregulation in NKp46^+^ ILC3, leading to acquisition of IFN-γ and an ILC1-like phenotype (16). In chronic tumor-associated inflammation, this conversion was typically gradual and became relatively stable over time. In mucosal infection and non-infectious inflammation models, IL-1β plus IL-23 drove high IL-17 and IL-22 output in the acute phase. In contrast, sustained IL-12 during chronic inflammation caused RORγt loss, T-bet upregulation, and conversion into IFN-γ-producing ILC1-like cells (17). These observations suggested that a shared ILC3 pool supported barrier repair early but contributed to tissue damage when inflammation persisted. IL-22 output was further tuned by an IL-23 and IL-2 STAT5 module. In colitis models, deletion of STAT5a and STAT5b in ILC3 reduced IL-23 and IL-2 induced STAT5 activation, decreased IL-22 production, increased susceptibility to Citrobacter rodentium infection, and compromised barrier integrity (18). These data indicate that IL-23 and IL-2, via STAT5 together with STAT3, set a minimal level of barrier-protective IL-22 production in ILC3. Non-immune cues also contribute. Specific strains of Akkermansia muciniphila enhance retinoic acid synthesis in dendritic cells, amplify IL-22 secretion by ILC3, and ameliorate dextran sulfate sodium–induced colitis through a retinoic acid–STAT3 axis (19). This microbiota–metabolism–ILC3 circuitry operates during the preneoplastic inflammatory phase of colorectal cancer (CRC). In this phase, microbiota composition, retinoic acid metabolism, and the cytokine milieu drive ILC3 toward either an IL-22^+^ reparative state or an IFN-γ^+^ proinflammatory state.

### 2.2 Transcriptional and epigenetic control

Upstream cytokines impose environmental pressure, but the ability of ILC3 to cross lineage boundaries is ultimately determined by their transcriptional and metabolic wiring. Immunometabolic studies indicate that aryl hydrocarbon receptor (AHR), mechanistic target of rapamycin (mTOR), hypoxia-inducible factor 1α (HIF-1α), and lipid metabolic pathways together provide the metabolic basis for ILC3 fate decisions. AHR ligands and specific fatty acid oxidation support RORγt-driven IL-22 production, whereas enhanced glycolysis, oxidative stress, and certain bile acid signals favor a shift toward IFN-γ and granulocyte–macrophage colony-stimulating factor (GM-CSF) (20). In a gut environment with fluctuating nutrients, microbial metabolites, and hypoxia, such metabolism-centered “soft regulation” allows ILC3 to span a functional spectrum from barrier maintenance to inflammatory amplification without immediate lineage conversion. Time is an additional layer in shaping plasticity. Nuclear receptors REV-ERBα and REV-ERBβ maintain chromatin accessibility at RORγt-associated loci, stabilize the ILC3 transcriptional program, and thereby limit conversion into IFN-γ^+^ ILC1-like cells (21). When circadian signals were perturbed, RORγt was more readily downregulated in ILC3 and T-bet was more readily induced under IL-12 stimulation. Type 1 biased responses were adopted. These findings expanded ILC3 plasticity from an acute cytokine-driven shift to a system-level program shaped by circadian control, epigenetic configuration, and environmental inputs. At the transcription factor level, the balance between T-bet and RORγt is a central hub of ILC3 fate control. In T-bet-deficient mice, NKp46^-^ ILC3 show a marked increase. These cells express higher levels of RORγt and IL-7 receptor but do not develop spontaneous colitis. These findings indicate that T-bet serves two roles: it acts as a brake on ILC3 accumulation and limits RORγt expression. By restraining these pathways, T-bet enables ILC3 to convert into ILC1-like cells when appropriate signals arrive (22). T-bet is not the only regulator, however. c-Maf constrains T-bet-driven programming in CCR6^-^ ILC3: c-Maf deficiency promotes acquisition of ILC1-like IFN-γ production, whereas c-Maf overexpression partially restores type 3 characteristics (23). In this context, c-Maf cooperates with RORγt and restrains T-bet, positioning ILC3 along a continuum between type 3 and type 1 states. Chromatin regulators further fix plasticity thresholds at the epigenetic level. BATF deficiency reduces chromatin accessibility at genes involved in MHC II–dependent antigen processing, limits the generation of MHCII^+^ ILC3, and disrupts the homeostasis of the microbiota and mucosal CD4^+^ T cells (24). Collectively, these data indicate that the likelihood of ILC3 converting into ILC1-like cells in response to inflammatory or tumor-derived signals is heavily influenced by the configuration of the RORγt–T-bet–c-Maf–BATF network within a given metabolic and temporal context.

### 2.3 Spatial and clinical heterogeneity

When these mechanisms are examined within CRC, they translate into recognizable changes in innate lymphoid cell composition in tumor tissues. Across animal models and patient samples, total ILC frequencies and subset distributions are frequently altered. In several cohorts, tumor tissues show reduced expression of ILC3 signature genes, together with increased expression of type 1–associated genes and enrichment of ILC1 or NK-like features, consistent with a dominant bias from ILC3 to ILC1 in the setting of tumor-driven chronic inflammation (25). This pattern aligns with sustained activation of the IL-12–T-bet axis and offers an innate immune explanation for high local IFN-γ levels and aggravated tissue damage. Phenotypic analyses of human tumor-infiltrating ILC further support this view. In small cohorts of resected CRC specimens, tumor tissues contain a higher proportion of CD127^+^ ILC1 and a lower proportion of NKp44^+^ natural cytotoxicity receptor (NCR)^+^ ILC3 compared with matched non-tumor mucosa, whereas NCR^-^ ILC3 are relatively preserved or modestly increased in some cases (26). These observations have led to the hypothesis that IL-12 and other type 1 mediators at the invasive front drive progressive conversion of NKp44^+^ ILC3 into ILC1-like cells, while residual NCR^-^ ILC3, influenced by the microbiota and TGF-β, preferentially produce IL-17. This “bidirectional imbalance” may characterize the innate lymphoid component of tumor-associated inflammation in CRC. Single-cell transcriptomic analyses refine this picture. In healthy intestine, ILC1, ILC3, and mixed ILC3/NK populations constitute the major innate lymphoid subsets, whereas CRC tissues harbor additional tumor-specific ILC1-like and ILC2 clusters. A fraction of ILC3 occupies an intermediate state between type 3 and type 1, and their cytokine and migration-related gene signatures correlate with tumor stage and clinical outcome (27). Thus, the shift from ILC3 to ILC1 in CRC appears to follow a continuous trajectory from NCR^+^ ILC3, through RORγt^+^T-bet^+^ intermediates, toward IFN-γ–high ILC1-like cells. The site along this trajectory where cells accumulate in a given region—tumor core, invasive margin, or distant mucosa—may determine whether that microenvironment is dominated by barrier repair or by inflammatory amplification and tissue damage.

### 2.4 Knowledge gaps: stratification, patient subsets, and treatment exposure

Despite advances in understanding the molecular basis of ILC3 plasticity and its outlines in CRC, studies that directly connect these mechanisms with clinical context remain limited, particularly along temporal, spatial, and patient stratification dimensions. Spatially, dynamic behavior of ILC3 in the intestinal mucosa is far less characterized than that of T cells. Intravital imaging has shown that, under steady-state conditions, villus-associated ILC3 are largely stationary, whereas during inflammation they acquire patrolling behavior, migrate between villi, secrete IL-22, and protect epithelial integrity; disruption of this behavior rapidly worsens barrier function (28). Comparable high-resolution observations in tumor tissues are largely lacking, leaving open questions about how ILC3 move and distribute within the tumor core, invasive front, and distant mucosa and how IL-22 fields evolve during tumor progression. From the perspective of patient and disease stratification, most clinical studies of ILC3 in CRC still rely on simple comparisons such as “CRC versus healthy controls” or “tumor versus adjacent tissue,” with little attention to microsatellite instability (MSI-H versus microsatellite stable), right- versus left-sided tumors, or microbiota-defined subtypes. Work in intestinal diseases has emphasized that the protective or pathogenic roles of ILC3 are highly context dependent and strongly influenced by disease subtype and microbial background: IL-12–driven conversion of NKR^+^ RORγt^+^ ILC3 into IFN-γ–producing ILC1 is linked to pathology in chronic colitis, whereas similar cells can support antitumor or anti-infective responses in other models (29). Without adequate stratification, simply quantifying ILC3 and ILC1 abundances is likely to yield conflicting or misleading conclusions.

Therapeutic exposure is an additional blind spot. Current evidence on how treatment reshapes ILC3 largely comes from inflammatory bowel disease and autoimmune settings. Narrative reviews highlight that ILC3 functions are modulated not only by short-term cytokine and microbiota changes but also by the formation of memory-like ILC3 after repeated inflammatory episodes, with lasting alterations in patrolling behavior, cytokine profiles, and tissue distribution (30). Encoded by the DEFB1 gene located on chromosome 8p23 (31), hBD-1 is a constitutively expressed antimicrobial peptide produced primarily by epithelial cells, including those lining the colonic mucosa. In the normal colon, hBD-1 is readily detectable in glandular epithelial cells, but its expression is frequently downregulated or lost in colorectal cancer (32). Functionally, hBD-1 serves as a ligand for CCR6, attracting immature dendritic cells and modulating immune responses (33). Importantly, recent data indicate that DEFB1-high CRCs are significantly less likely to exhibit MSI-H/dMMR or high TMB, and are associated with reduced CD8^+^ T cell infiltration and worse overall survival (34). Against this background, we argue that in CRC, chemotherapy, radiotherapy, and immune checkpoint blockade are all expected to remodel gut ILC3, pushing some populations toward stronger type 1 inflammation while driving others into functional exhaustion or impaired migration. However, prospective cohorts that track ILC3 plasticity across defined treatment time points are virtually absent, and there is almost no work integrating these dynamics with MSI status, microbiota configurations, and DEFB1 expression. These gaps help explain why there is still no consensus on whether ILC3 in CRC act as uncontrolled drivers, passive bystanders, or context-dependent regulators that assume different roles in distinct patients and disease stages. In subsequent sections, we focus on their antigen presentation–like functions and on the coupling between ILC3 plasticity and the epithelial–microbiota–human beta-defensin 1 axis, aiming to move the discussion from simple “good versus bad” dichotomies toward a more precise description of where, in whom, and in which directions this network becomes unbalanced.

## 3 Antigen-presenting functions of ILC3

Antigen presentation–like activity provided a second layer by which ILC3 influenced adaptive immunity beyond cytokine secretion. Here, evidence organized around MHC II-dependent immune gating of microbiota-directed CD4^+^T cell responses and downstream IgA targeting. Technical and conceptual issues that affected interpretation in colorectal cancer settings were also summarized.

### 3.1 MHC II-dependent immune gating

Beyond cytokine secretion, intestinal ILC3 stably express major histocompatibility complex class II (MHC II). RORγt^+^ ILC3 in Peyer’s patches, mesenteric lymph nodes, and the lamina propria localize near CD4^+^ T cell–rich regions. They also process luminal microbial and dietary antigens for loading onto MHC II (35, 36). By modulating co-stimulatory and co-inhibitory molecules in an inflammation-dependent manner, they influence local Th17, regulatory T (Treg), and effector/memory T cell responses. In this context, ILC3 are often described as “non-classical” antigen-presenting cells (APC), specialized in presenting commensal and environmental antigens and in fine-tuning T cell activation thresholds and lineage bias rather than driving strong primary T cell expansion (37, 38). Comparative analyses indicated that conventional dendritic cells and ILC3 both expressed high levels of MHC II and were able to form immune synapses with T cells, but their functional profiles differed. In mouse models, conventional dendritic cells supported rapid antigen uptake, processing, and presentation to naïve T cells (39). In contrast, studies in mice have shown that ILC3 are long-lived tissue-resident cells (40) that displayed lower-density peptide MHC II complexes and provided milder co-stimulation (41). Both mouse and human studies have demonstrated that IL-22 and GM-CSF are produced by ILC3 and are used to modulate pre-existing T cell pools in mucosal tissues (39, 42). In humans, the identity of MHCII^+^ RORγt^+^ innate cells is more heterogeneous. Single-cell transcriptomic and chromatin accessibility studies have identified rare MHCII^+^ RORγt^+^ “R-DC-like” cells in adult and fetal mesenteric lymph nodes that co-express DC2 markers and ILC3-associated transcriptional programs, localize to T cell zones, and exhibit mature APC function (35). These findings indicate that the RORγt^+^MHCII^+^ phenotype encompasses multiple lineages and developmental origins. Moreover, local factors such as oxygen tension can limit the antigen-presenting capacity of bona fide ILC3: in the pregnant uterus and placenta, hypoxia downregulates MHC II on NCR^+^ ILC3 and reduces their potential to regulate T cells and NK cells (36). Overall, MHCII expression by ILC3 should be viewed as part of a broader spectrum of RORγt^+^MHCII^+^ APC populations whose function is shaped by non-immune environmental cues as well as inflammatory signals.

### 3.2 IgA focusing and microbiota shaping

Secretory immunoglobulin A (sIgA) is the most abundant antibody isotype in intestinal secretions in both humans and mice. It limits pathogen adhesion, promotes immune complex clearance, and coats commensal bacteria, thereby restraining bacterial translocation across the epithelium and shaping microbiota composition in spatial and metabolic terms. sIgA is generated through T cell–independent pathways in isolated lymphoid follicles and through T cell–dependent germinal center reactions in organized structures such as Peyer’s patches, where T follicular helper (Tfh) cells, follicular dendritic cells, and cytokines coordinate class-switch recombination and affinity maturation (43). Any cell type that modifies Tfh activity, antigen focusing, or germinal center dynamics can thus indirectly alter IgA specificity. For instance, ILC3 within intestinal draining lymph nodes regulate Tfh-B cell interactions through MHCII-dependent antigen presentation, thereby limiting mucosal IgA responses to commensal bacteria (44, 45). Intestinal IgA responses are dynamic rather than static. In mice, IgA secretion by plasma cells, the proportion of IgA-coated bacteria, and transcriptional activity of associated metabolic pathways exhibit circadian oscillations, driving rhythmic changes in the composition and localization of commensal communities (45). These “antibody–microbiota” rhythms depend on sustained metabolic support and upstream immune signals. Multi-omics analyses implicate IL-22, GM-CSF, and the metabolic state of mucosal dendritic cells as key determinants of IgA rhythmic amplitude, with a substantial fraction of these upstream cues originating from ILC3. Reviews summarizing ILC–microbiota–IgA interactions describe a multi-layered feedback network: microbial signals induce IL-23 and IL-1β production by dendritic and epithelial cells, which activate ILC3 to secrete IL-22 and GM-CSF (46). These cytokines act directly on epithelial cells to induce antimicrobial peptides and mucus and indirectly on mucosal dendritic cells and the Treg/Tfh axis to adjust IgA targeting profiles. In parallel, pattern-recognition receptor–APC–lymphocyte circuits allow microbiota composition to reprogram local IgA responses and inflammatory thresholds, which in turn feedback on microbial ecology (47). Within this network, APC-like ILC3 occupy an upstream position: via MHC II and co-stimulatory molecules they modulate T cell–dependent IgA responses, and via IL-22 they influence how the epithelium responds to IgA-coated bacteria, enabling IgA to function not only as a barrier but also as a shaper of microbial communities.

### 3.3 Methodological caveats

Early murine studies suggested that MHC II–expressing ILC3 were required for mucosal tolerance (45, 48). Loss of MHC II in ILC3 was associated with exaggerated responses to commensals, reduced regulatory T cells, and spontaneous colitis (45, 49, 50) Defects were mainly revealed under high-dose infection and conditions requiring rapid restoration of epithelial homeostasis, where tissue damage and mortality were increased (51). These findings suggest that ILC3 primarily reinforce tolerance and barrier protection when sufficient T cell-derived IL-22 is available. In this model, ILC3 do not act as indispensable initiators of T cell activation. A stronger revision emerged with the identification of Thetis cells, a RORγt and MHC II positive antigen-presenting population enriched in neonatal mesenteric lymph nodes (52). Selective MHC II loss in distinct antigen-presenting compartments produced different outcomes. MHC II deficiency in Thetis cells, but not in ILC3, disrupted microbiota-induced peripheral regulatory T cell generation and caused colitis and loss of tolerance (52). These data argue that, during the early life tolerance window, transient Thetis cells—not numerically stable ILC3—are the main inducers of “do not attack commensals” programs in CD4^+^ T cells. Conceptual and technical issues further complicate interpretation of ILC3–MHCII studies. Across studies, different exclusion markers, NCR-based definitions, and criteria for separating lymphoid tissue inducer cells from ILC3 were applied. As a result, the term MHC II positive ILC3 often referred to non-identical cell populations across experiments (53). The presence of RORγt^+^MHCII^+^ DC-like cells in human samples adds another layer of heterogeneity (54). Thus, apparent contradictions—where one study finds ILC3-mediated antigen presentation crucial and another deems it dispensable—may reflect differences in cell definitions and technical approaches as much as true biological divergence. Cross-tissue analyses also emphasize that most functional data on ILC3–MHCII derive from the mouse intestine; in organs such as the skin and lung, evidence is largely indirect, and in human tissues *in situ* imaging and functional dissection of ILC3–T cell interactions remain scarce (55). Under these circumstances, extending any categorical conclusion about ILC3–MHCII being essential or non-essential directly to CRC is premature.

### 3.4 CRC-related hypotheses

In the specific context of CRC, MHCII^+^ ILC3 may contribute to immune network imbalance at several levels. Drommi et al. proposed an ILC3–microbiota–tumor axis. Under homeostatic conditions, ILC3 maintain epithelial integrity and limit bacterial translocation via IL-22, while their MHCII-dependent interactions with T and B cells support controlled, commensal-focused immune responses (53). In chronic inflammation and persistent carcinogenic signaling, ILC3 dysfunction or exhaustion, together with dysbiosis and sustained IL-23 activation, favors expansion of pro-tumor bacteria, barrier disruption, and accumulation of immunosuppressive cells, thereby establishing a protumor microenvironment. Within this framework, dysregulation of the ILC3–MHCII axis may reflect both failure to restrain inappropriate commensal responses and defocusing or tolerization of antitumor T cell responses. Focusing on APC-like ILC3, Calabrò et al. proposed that MHCII-expressing ILC3 in tumors are highly plastic APC whose antigen presentation, in different co-stimulatory or co-inhibitory contexts, can skew CD4^+^ T cells toward effector or regulatory fates (56). When MHC II levels are maintained and co-stimulatory molecules such as OX40L and CD30L are expressed, ILC3 in tumor-draining lymph nodes may help focus T cell responses on carcinogenic bacteria and tumor-associated antigens, thereby supporting both IgA production and cytotoxic T cell activity (57, 58). Conversely, in a microenvironment shaped by hypoxia, TGF-β, and metabolic reprogramming, ILC3 may display low MHC II or present antigen in the absence of adequate co-stimulation, favoring anergic or regulatory CD4^+^ T cells and facilitating immune escape (45, 59).

From a broader gastrointestinal perspective, Hao et al. integrated data on ILC3, microbiota, and gut diseases and concluded that ILC3 imbalance is a common feature of infectious diarrhea, inflammatory bowel disease, CRC, and irritable bowel syndrome (60). They emphasized that MHCII^+^ ILC3 together with the IL-22/IL-17 axis form a core component of a “barrier–inflammation–microbiota” triangle: when ILC3 are numerically or functionally impaired, barrier disruption, prolonged pattern-recognition receptor activation, and microbiota remodeling amplify one another, promoting chronic inflammation and cancer risk, whereas in early or localized lesions residual MHCII^+^ ILC3 may offer a relatively plastic entry point for restoring mucosal homeostasis and microbiota composition. Incorporating IgA into this picture suggests a potential tumor-suppressive loop. In an azoxymethane/dextran sulfate sodium–induced colitis-associated CRC model, Tang et al. showed that mice lacking MZB1 have reduced intestinal IgA, markedly altered microbiota, more severe colitis, and increased tumor number and size; supplementation with exogenous monoclonal IgA partially reversed these changes, attenuating inflammation and limiting tumor development (61). This work demonstrates that maintaining sufficient, appropriately targeted IgA is a key condition for resisting inflammation-driven CRC. Taken together with the role of ILC3 as upstream regulators of T cell–dependent IgA, a working hypothesis emerges: in the CRC microenvironment, reduced frequency of MHCII^+^ ILC3 or a shift toward antigen presentation with inadequate co-stimulation may not only bias CD4^+^ T cells toward low-responsiveness or tolerance but also defocus IgA programming, promoting a “low IgA–high inflammation–high tumor burden” state analogous to that seen in MZB1-deficient mice. Conversely, in tumors or precursor lesions enriched in APC-competent ILC3 capable of productive interactions with Tfh cells, local and systemic IgA responses may be better directed against pro-tumor microbiota, forming an “ILC3–T cell–IgA” circuit with tumor-suppressive potential.

## 4 The epithelium–microbiota–hBD-1 axis

**Figure 1:**
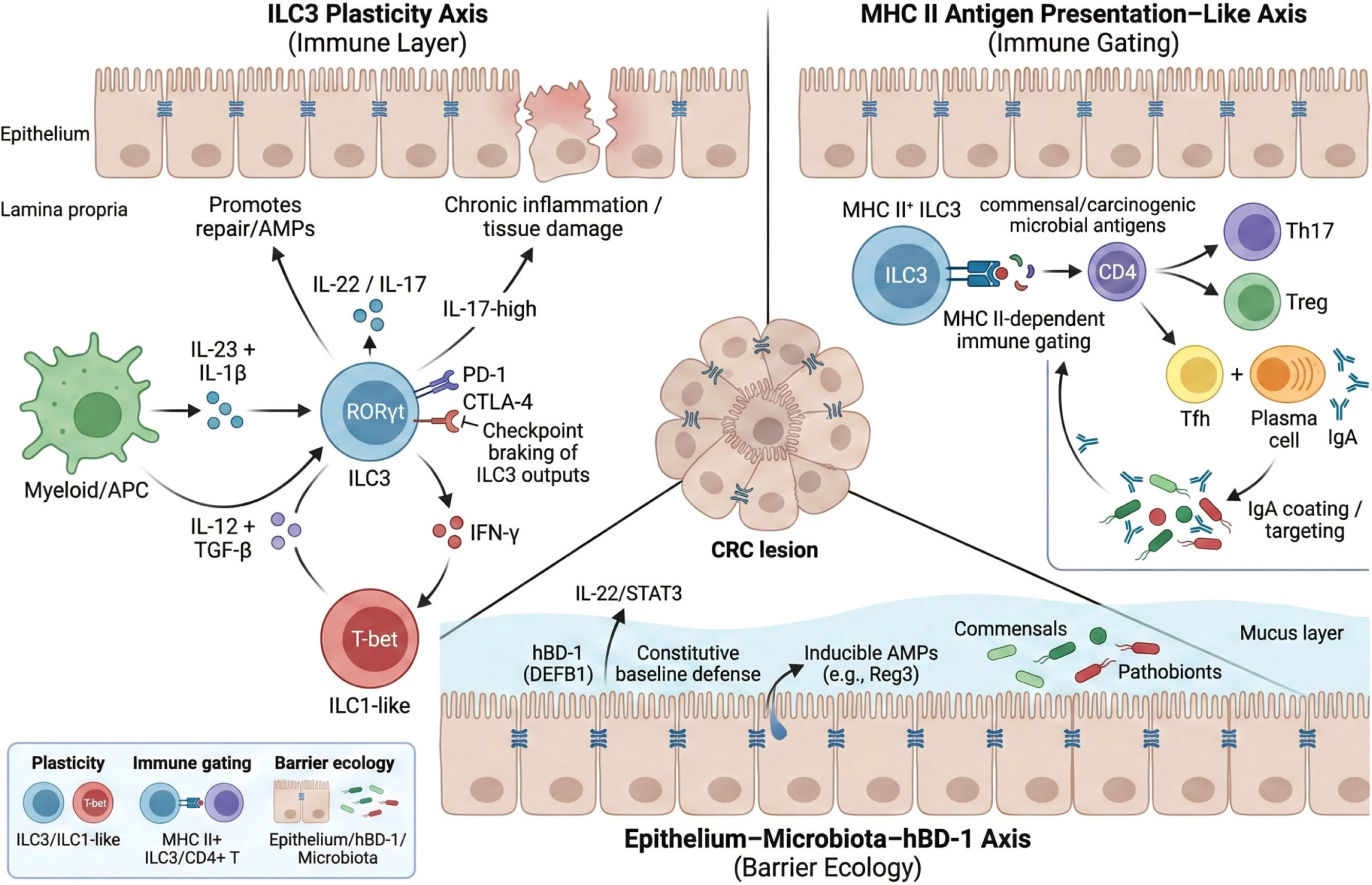
ILC3-centered tri-directional network and imbalance loops in colorectal cancer. Schematic of a coupled mucosal network in CRC linking (i) ILC3 plasticity (IL-23/IL-1β-driven IL-22/IL-17 programs versus IL-12/TGF-β-driven ILC1-like IFN-γ states), (ii) MHC II–dependent immune gating by APC-like ILC3 shaping CD4+ T-cell polarization and Tfh–IgA targeting, and (iii) epithelial barrier ecology integrating constitutive hBD-1 (DEFB1) with IL-22–STAT3-induced repair and antimicrobial programs. Key CRC-associated imbalance patterns are highlighted, including an IL-22–OSM–STAT3 feed-forward circuit, ILC3-to-ILC1 skewing, weakened antigen focusing with IgA misprogramming, and a “low hBD-1” loop amplifying PRR–IL-23 inflammatory pressure.

Epithelial antimicrobial programs set the baseline noise level of the mucosal ecosystem and constrained dysbiosis. This section focused on DEFB1 and hBD-1 as a constitutive barrier component and integrated its regulation with IL-22-dependent repair programs and microbial selection pressure. The goal was to connect epithelial barrier status to shifts in ILC3 states and microbiota composition (**Figure 1**).

### 4.1 Overview of hBD-1

Human beta-defensin 1 (hBD-1), encoded by DEFB1, is a member of the β-defensin family that is constitutively produced by epithelial cells of the intestine, urinary tract, and respiratory tract. Unlike many inducible α/β-defensins that are sharply upregulated during infection or overt inflammation, hBD-1 is expressed at low but stable levels under homeostatic conditions and provides broad-spectrum activity against Gram-negative and Gram-positive bacteria, as well as certain fungi and viruses (62). Recent work has emphasized that defensins are not merely membrane-disrupting “mini-antibiotics” but also act as chemotactic factors and modulators of host receptors and signaling pathways, thereby contributing to mucosal immune homeostasis, barrier repair, and long-term shaping of host–microbiota relationships (62). Compared with other β-defensins such as hBD-2 and hBD-3, hBD-1 has a lower activation threshold. In multiple epithelial tissues, hBD-1 mRNA and protein are detectable in the absence of overt inflammation, whereas hBD-2 and hBD-3 are strongly induced by microbial-associated molecular patterns, Toll-like receptor signaling, or pro-inflammatory cytokines (63). This pattern supports a role for hBD-1 as a “baseline guard” during apparent homeostasis, continuously limiting the depth of colonization and translocation of potential pathogens without dramatically perturbing the microbial ecosystem. In line with this concept, tissue studies in patients with bladder cancer have shown that tumor-associated microbiota composition changes markedly under different antibiotic regimens, yet hBD-1 expression is consistently upregulated across treatment groups, whereas inducible hBD-2/hBD-3 responses vary in a more drug-specific manner (63). Beyond direct antimicrobial activity, hBD-1 appears to exert broader immunoregulatory effects. In non-segmental vitiligo, circulating hBD-1 levels are reduced compared with healthy controls, and specific DEFB1 polymorphisms associate with disease risk, suggesting that hBD-1 may influence susceptibility through anti-inflammatory or immunomodulatory mechanisms (64). In the intestinal epithelium, a physiologically hypoxic environment, stabilization of hypoxia-inducible factor 1α (HIF-1α) upregulates multiple barrier-related molecules, strengthens tight junctions, and induces protective cytokines such as IL-22 and IL-10, thereby alleviating experimental colitis (65).

### 4.2 Bidirectional dialogue with the microbiota

From a microbial ecology perspective, the colon is a dense and rapidly evolving ecosystem in which host defense strategies rarely aim at simple elimination of “invaders,” but rather at continuously adjusting selective pressures so that certain communities are favored while others are constrained (66). In this setting, a constitutively expressed peptide such as hBD-1, whose activity can be modulated by local pH, redox state, and nutrient availability, is well suited to act as a fine-grained selector. By selectively restricting particular taxa, it helps set the “baseline diversity” of the mucosal ecosystem and prepares the ground for subsequent fine-tuning by IgA and cellular immunity (66). The microbiota, in turn, is not a passive target of defensins. After perturbations such as antibiotics, dietary shifts, or disease flares, microbial communities display distinct patterns of rebound, and the host antimicrobial peptide repertoire, including β-defensins, is a major determinant of whether recovery converges toward a healthy configuration or stabilizes in a dysbiotic state (67). Prolonged low defensin expression facilitates durable colonization of pathobionts at the mucosal surface and establishment of a new, potentially harmful equilibrium; conversely, very strong transient upregulation may suppress pathogens but also reduce overall diversity and resilience (68). Evidence from non-intestinal diseases supports a similar logic. In children with obesity and metabolic dysfunction–associated steatotic liver disease (MASLD), serum levels of α-defensins 5 and 6, together with markers of intestinal barrier disruption, correlate with indices of gut inflammation, microbiota alterations, and liver injury (69). Although this work focuses on α-defensins and the gut–liver axis, it reinforces the concept that epithelial antimicrobial peptides reshape barrier permeability, local inflammation, and metabolic pathways, and thereby indirectly sculpt the microbiota. For hBD-1, steady-state expression combined with sensitivity to the local milieu makes it a plausible long-term “farmer” of the intestinal microbiota: preventing outgrowth of “weeds” without indiscriminately clearing the field.

### 4.3 hBD-1/DEFB1 in CRC

In colorectal cancer (CRC), the role of DEFB1 becomes more complex. *In vitro* studies indicate that hBD-1 can inhibit mTOR signaling, activate autophagy, and directly impair the survival and clonogenic capacity of colon cancer cells, consistent with tumor-suppressive potential. However, large-scale molecular pathology data tell a more nuanced story. In an analysis of more than 14,000 patients with CRC, tumors were stratified by DEFB1 RNA expression and displayed distinct molecular and clinicopathological patterns: High DEFB1 expression occurred more frequently in left-sided and rectal cancers. It also associated with specific consensus molecular subtypes and higher rates of APC mutations. In contrast, DEFB1-low tumors enriched for mutations in genes such as BRAF, SMAD4, and RNF43 (34). Notably, DEFB1-high tumors showed an immune profile characterized by increased expression of immune checkpoint genes, relatively limited CD8^+^ T cell infiltration, and a higher proportion of M2-like macrophages, along with slightly worse overall survival (34). These findings suggest that DEFB1 upregulation does not uniformly indicate stronger antimicrobial or antitumor defense. In some backgrounds, it may represent an adaptive response to chronic barrier stress and dysbiosis within an immune-suppressed microenvironment, where the intrinsic antimicrobial and immunoregulatory actions of hBD-1 are outweighed by checkpoint pathways, metabolic competition, and suppressive cytokines. DEFB1/hBD-1 in CRC is therefore likely a context-dependent player: potentially tumor-suppressive in early or localized disease, but in established tumors serving mainly as a biomarker of barrier pressure and immune imbalance rather than an effective brake on progression.

### 4.4 Indirect interplay with ILC3

The most natural entry point for integrating hBD-1 into the “ILC3–epithelium–microbiota” network is IL-22. This cytokine is abundantly produced by ILC3 and specific CD4^+^ T cell subsets. Microbiota- and diet-derived tryptophan metabolites activate the aryl hydrocarbon receptor (AhR) in immune cells, promoting IL-22 production; IL-22 then signals through STAT3 in epithelial cells to support proliferation and repair, enhance mucin and antimicrobial peptide (for example, Reg3 family) expression, strengthen tight junctions, and limit bacterial translocation (67). In experimental colitis, enhancement of the AhR–IL-22 axis correlates with improved barrier integrity, restructuring of the microbiota, and attenuation of inflammation, whereas inhibition of this pathway impairs epithelial repair and sustains chronic inflammation (67). Genetic modulation of IL-22 regulation further underscores its protective potential. Deletion of IL-22 binding protein (IL-22BP) prolongs and intensifies IL-22 signaling in colonic epithelium, leading to increased antimicrobial peptide and mucus production, greater resistance to pathogenic colonization, and reduced barrier damage and inflammation, without causing a collapse in microbiota diversity (70). Superimposing this IL-22 axis onto hBD-1 biology suggests a layered mode of indirect interaction: homeostatic IL-22 supports inducible defenses while hBD-1 provides static defense; acute IL-22 drives coordinated upregulation of defensins and repair, with hBD-1 helping to prevent bacterial translocation; in chronic inflammation and preneoplastic CRC, failure of the AhR–IL-22 pathway together with DEFB1 repression may weaken barrier integrity and promote a carcinogenic microbiota. Thus, ILC3–IL-22 and hBD-1/DEFB1 act as two convergent arms of the epithelial barrier program; persistent imbalance in either arm may drive the triad toward inflammation, dysbiosis, and CRC progression.

## 5 Tri-directional crosstalk in CRC

Multiple feedback loops among ILC3 state transitions, barrier integrity, and microbial ecology were repeatedly linked to tumor-promoting inflammation. In this section, we assembled these interactions into recurrent loop motifs. These motifs captured how a low-noise barrier state was lost and how chronic inflammation persisted. The resulting framework supports stratification and intervention design.

### 5.1 IL-22/OSM/STAT3 axis

In acute mucosal injury, the IL-22/STAT3 axis is predominantly protective. IL-22 rapidly activates STAT3 in intestinal epithelial cells, inducing anti-apoptotic and pro-proliferative genes, tight junction components, and mucus-associated molecules to promote rapid barrier repair. In colorectal cancer (CRC) cell and organoid models, however, IL-22 stimulation also upregulates ICAM-1 and oncogenesis-related genes such as NNMT and CEA, enhancing tumor cell migration and proliferation via STAT3-dependent transcriptional programs (71). Under chronic inflammatory and chemotherapeutic pressure, IL-22 can further support tumor adaptation. Prolonged IL-22 exposure increases expression of stemness-associated factors such as SOX2, OCT4, and NANOG, promotes spheroid formation, and activates EGFR/AKT/ERK signaling, thereby decreasing sensitivity to oxaliplatin-induced cytotoxicity (72). A recent *in vivo* study defined an IL-22/oncostatin M (OSM)/STAT3 loop in colitis-associated cancer: ILC3-derived IL-22 upregulates OSM receptor (OSMR) in epithelial cells through STAT3, sensitizing them to OSM; immune cell–derived OSM then cooperates with IL-22 to sustain high STAT3 activity and induce an “inflammatory adaptation” program involving chemokines, matrix remodeling, and epithelial transformation genes, whereas epithelial OSMR deletion or pharmacological OSM blockade attenuates colitis and colitis-associated tumorigenesis (72). OSM itself displays similar duality. As a member of the IL-6 family, it contributes to wound healing and tissue regeneration but is also associated with therapy-resistant inflammation and tissue destruction. Via OSMR/gp130, OSM activates JAK/STAT3, MAPK, and PI3K/AKT pathways to promote cell proliferation, survival, angiogenesis, and recruitment of inflammatory and myeloid-derived suppressor cells in chronic inflammatory diseases and solid tumors, including CRC (73, 74). Taken together, microbiota-driven, long-lasting activation can rewire an initially reparative IL-22/OSM/STAT3 circuit into a pro-tumor module that supports epithelial hyperproliferation and a chronically inflamed, immunologically imbalanced microenvironment.

### 5.2 ILC3 to ILC1 shift and checkpoint control

ILC3 sit on a continuum with ILC1, and their relative proportions shift in solid tumors. Reviews of ILC in tumor immunity indicate that in several cancers, including CRC, a bias toward ILC1 associates with disease stage and response to immunotherapy, with higher local interferon-γ (IFN-γ), increased tissue damage, and reduced IL-22-mediated barrier protection, suggesting that ILC3-to-ILC1 reprogramming reflects a broader deviation of the mucosal immune network (75). Checkpoint molecules act as critical brakes on this plasticity. In the gut, a subset of ILC3 expresses PD-1, which modulates metabolic homeostasis and the balance between protective and proinflammatory outputs rather than simply suppressing function, thereby influencing ILC3 plasticity (76). Other checkpoints show comparable effects. CTLA-4 is expressed on ILC1, NCR^-^ ILC3, and NCR^+^ ILC3; its deletion or blockade within the innate compartment alone triggers an IFN-γ/IL-17-skewed transcriptional program and exacerbates colitis and checkpoint inhibitor–associated colitis, even in the absence of T cell involvement (77). In an autoimmune nephritis model, PD-1 controls endocytosis of the IL-23 receptor in ILC3, thereby tuning JAK2/STAT3/RORγt/IL-17A signaling and their contribution to tissue injury versus repair (78). These findings imply that in CRC-associated chronic inflammation and under immune checkpoint blockade, the combined effects of ILC3-to-ILC1 skewing and reconfigured PD-1/CTLA-4 signaling may determine whether IL-22-mediated protection is preserved or lost and whether IFN-γ-driven damage and treatment-related toxicity are amplified.

### 5.3 Antigen presentation and IgA misprogramming

On the adaptive side of the tri-directional model, IgA is a central link between mucosal immunity and microbiota structure. In a single-cell and spatial transcriptomic study of 42 patients with CRC, Fusobacterium nucleatum–positive tumors showed impaired development of IgA plasma cells and reduced production of secretory IgA, leading to diminished mucosal IgA coating, higher intratumoral bacterial loads, persistent inflammatory signaling, and poorer clinical outcomes (79). New high-throughput approaches are refining how IgA targets are mapped at the strain level. A next-generation IgA-SEQ platform that combines magnetic bead–based separation with plate formats allows sorting of large numbers of IgA-coated bacteria under anaerobic conditions and subsequent metagenomic profiling and culture. Application to stool samples from healthy individuals and patients with inflammatory bowel disease revealed that, at steady state, roughly 10–50% of bacteria are IgA-coated, rising to about 90% during inflammation, and that highly IgA-coated taxa often overlap with the immunostimulatory core of disease-driving microbes (80). Immunological reviews of CRC emphasize that microbial influence on T cells operates through a multilayered cascade: pathogen-recognition receptor–mediated innate sensing, antigen presentation patterns set by APC (including APC-like ILC3), T follicular helper–driven germinal center reactions, and IgA class switching and affinity maturation (81). Perturbation at any step can convert a controlled “defensive” inflammatory response into a carcinogenic one. In CRC lesions where MHCII^+^ ILC3-mediated antigen presentation is weakened or biased and bacteria such as F. nucleatum actively impair IgA production, the system may evolve toward a composite state in which regulation of commensal reactivity is lost while IgA targeting of truly oncogenic microbiota is insufficient, facilitating microbe-driven tumor-promoting inflammation.

### 5.4 The low hBD-1 loop

At the outer layer of the imbalance map, DEFB1/hBD-1 can be envisaged as a progressively downregulated antimicrobial barrier. In CRC cells, hBD-1 has been shown to inhibit AKT/mTOR signaling, increase Beclin-1 and LC3-II/I expression, induce autophagy, and limit proliferation; a recent translational study integrated this effect into a non-coding RNA network, demonstrating that circRNA HAS-CIRCpedia-5280, acting as a sponge for miR-4712-5p, modulates AKT/mTOR activity upstream of hBD-1. Overexpression of this circRNA enhances CRC cell proliferation and migration and suppresses autophagy, whereas exogenous hBD-1 restores autophagy and growth control (82). In parallel, chronic dysbiosis drives tumorigenesis through both direct and indirect routes. Reviews of the microbiota–CRC axis highlight that specific carcinogenic bacteria generate toxins, genotoxic metabolites, and reactive oxygen species that damage epithelial DNA, while broader community shifts activate Toll-like receptors, NOD-like receptors, and other pattern-recognition receptors to induce IL-1β, tumor necrosis factor-α, IL-23, and related inflammatory cascades, sustaining STAT3 and other oncogenic pathways (83, 84). If DEFB1 expression is chronically low or functionally constrained, reduced defensin-mediated selection allows carcinogenic or highly immunostimulatory taxa to colonize the mucosal surface more stably and in closer proximity to the epithelium, further amplifying pattern-recognition signaling. IL-23 occupies a central position at the intersection of inflammation and neoplasia in the gut. It is a key driver of the Th17/IL-17 axis and a critical survival and functional signal for ILC3, and sustained IL-23 exposure aggravates intestinal inflammation and accelerates colitis-associated CRC in experimental models (85). Work on the gut microbiota–immune axis in CRC indicates that dysbiotic communities activate PRRs not only on classical myeloid cells but also on ILC3, which, under persistent IL-23 and IL-1β stimulation, tend to shift from IL-22-dominant, barrier-protective states toward IFN-γ/IL-17-high inflammatory phenotypes that synergize with barrier disruption to drive tumor-promoting inflammation (86). Taken together, these lines of evidence support a “low hBD-1 loop” in CRC: genetic, epigenetic, or non-coding RNA–mediated suppression of DEFB1 weakens the antimicrobial peptide barrier; carcinogenic and immunostimulatory bacteria gain a foothold at the mucosal surface; chronic activation of pattern-recognition pathways elevates IL-23, IL-1β, and IL-6; ILC3 are pushed toward ILC1-like or exhausted states with loss of IL-22-mediated protection; and progressive barrier damage, microbiota imbalance, and chronic inflammation converge on STAT3, EGFR/ERK, and related pathways to support tumor initiation and progression.

## 6 Measurable indicators and therapeutic targets

To translate a coupled network into measurable and actionable variables, biomarkers and therapeutic levers were organized along the same three axes. Minimal biomarker sets were proposed to capture ILC3 state, antigen focusing, and epithelial barrier pressure, and candidate intervention routes were summarized. Timing and combination logic were emphasized to reduce the risk of pushing the network toward new unstable states.

### 6.1 Multilayer biomarker panels

The preceding sections outline a complex immune network; a biomarker panel is, in essence, an attempt to compress this network into a set of clinically accessible readouts. The key question is which cell populations, molecules, and transcriptional features in a tissue section, a few milliliters of blood, or a microbiome profile can reliably reflect the state of ILC3 plasticity, MHCII-dependent antigen presentation, and the epithelium–microbiota–hBD-1 axis. At the single-cell level, the lineage and activation states of tumor-infiltrating ILC represent a highly informative layer. In the AOM–DSS model, single-cell RNA sequencing has shown that ILC3 follow a trajectory from RORγt^+^ ILC3 toward regulatory ILC (ILCreg), accompanied by redistribution of ILC1 and ILC2 subsets; the proportions and functional states of these clones correlate closely with tumor burden and progression speed (87). This suggests that, in human CRC, parameters such as the intratumoral ILC3/ILC1 (and ILCreg) ratio and the RORγt/T-bet expression gradient could serve as cellular markers of “how far” ILC3 plasticity has been pushed, quantifiable by multiparameter flow cytometry in combination with spatial transcriptomics. From a panoramic immune-infiltration perspective, integrated transcriptomic analyses across multiple cohorts have demonstrated that the extent and pattern of innate and adaptive immune cell infiltration in CRC tissue strongly predict postoperative recurrence risk and that such “immune infiltration signatures” outperform traditional molecular classifications such as CMS in several datasets (88). More refined analyses further suggest that ratios of specific subpopulations capture network deviation more sensitively than absolute cell counts; for example, in MSI-high tumors, the ratio of exhausted to tissue-resident memory T cells can outperform single-population metrics in predicting response to immune checkpoint blockade (89). Applying this logic to ILC3 implies that ratios such as ILC3/ILC1, MHCII^+^ ILC3/total ILC3, or the proportion of PD-1^+^/CTLA-4^+^ cells within ILC3 could be incorporated into composite immune scores alongside T cell and myeloid cell ratios.

Circulating markers add temporal resolution and practicality for serial monitoring. Studies of Th17/Treg-associated transcription factors have shown that peripheral blood mRNA levels of RORγt and Foxp3, and the corresponding Th17/Treg ratio, correlate with tumor size, TNM stage, and lymph node metastasis, and independently associate with CRC-specific mortality; elevated IL-17 and IL-22 concentrations typically indicate more active inflammation and more aggressive disease (90). A multiplex Luminex-based analysis of 40 circulating cytokines found that patients with advanced CRC exhibit increased levels of multiple chemokines, IL-2, IL-4, and myeloid polarization factors, together with reduced IFN-γ, forming a “cytokine cloud” that tracks with tumor stage and diameter (91). A review of “old and new” blood biomarkers for CRC explicitly lists IL-17A, IL-22, and TGF-β among cytokines with prognostic relevance, complementing traditional markers such as CEA, CA19-9, and circulating tumor DNA to provide a serum-level readout of the inflammation–tumor–immune triangle (92). Taken together, these observations support a multilayer biomarker framework. At the tissue level, the ILC3/ILC1 ratio, the frequency of MHCII^+^ ILC3, the RORγt/T-bet axis within ILC and T cells, the balance between exhausted and memory T cells, and MSI status combined with tertiary lymphoid structure density could reflect the state of local cellular immunity. At the circulating level, serum IL-22, IL-17, and related cytokines, Th17/Treg-associated transcription factors, and chemokines linked to the ILC3–IL-23 axis would capture systemic inflammatory and ILC-driven signals. At the epithelial/microbiota level, expression of DEFB1 and other antimicrobial peptides, together with the abundance of immunostimulatory bacterial strains and their IgA-coating profiles, would mirror barrier pressure and microbiota–immune interactions. In such a composite panel, no single marker is overinterpreted; each serves as one projection of the underlying “ILC3 plasticity–antigen presentation–barrier” state, allowing clinicians to infer the rough shape of a complex network from a finite set of measurements.

### 6.2 Tuning ILC3 states

To actively reshape ILC3 behavior in CRC, the most intuitive strategy is to modulate their plasticity—not to eliminate ILC3, but to adjust where they reside along the ILC3/ILC1/ILCreg spectrum. Recent mechanistic work has identified increasingly precise control points. Analysis of cis-regulatory elements at the Rorc locus has revealed that the CNS9 region is a critical switch for ILC3 lineage stability: deletion of CNS9 markedly reduces RORγt protein levels, impairs ILC3 numbers and function in the small intestine, and predisposes cells to drift away from the type 3 lineage (93). This highlights that, beyond the canonical RORγt/T-bet axis, specific enhancers and super-enhancers are integral structural determinants of ILC3 fate and potential targets for future gene- or small-molecule–based interventions. From a druggability perspective, the IL-12/IL-23 cytokine family remains the most immediate entry point. Clinical trials have established that antibodies targeting the IL-12/23 p40 subunit or the IL-23 p19 subunit (for example, ustekinumab, mirikizumab, risankizumab) achieve durable clinical responses and mucosal healing in a substantial proportion of patients with moderate-to-severe inflammatory bowel disease, with favorable safety profiles in multiple phase II/III studies (94). Mucosal transcriptomic analyses of ulcerative colitis patients treated with mirikizumab further showed downregulation of IL-1β, OSMR, and other genes associated with resistance to anti-TNF and JAK inhibitors, and a broader reversion of the “high-tension IL-23/OSM/STAT3” gene signature in inflamed tissue (95). Although generated in non-malignant cohorts, these data suggest that, within the shared intestinal ecosystem, carefully calibrated attenuation of IL-23 signaling could shift ILC3 away from IL-17/IFN-γ-high, proinflammatory and pro-tumor states toward IL-22-dominant, barrier-supportive programs, provided that dose, timing, and tumor burden are judiciously balanced.

“Ecological” strategies based on nutrition and metabolism are particularly attractive for chronic and pre-neoplastic settings. Reviews of micronutrients and ILC biology note that vitamins A and D and certain dietary aryl hydrocarbon receptor (AhR) ligands modulate ILC development and function, contributing to mucosal barrier maintenance: the vitamin A/retinoic acid axis supports ILC residency in the intestine and IL-22 production, whereas vitamin D is associated with anti-inflammatory ILC phenotypes (96). A dedicated review on AhR emphasizes that many diet-derived ligands promote anti-inflammatory programs and enhance ILC3–IL-22–antimicrobial peptide pathways, while some environmental toxic ligands can adversely affect Th17/Treg balance and ILC functions (97). These insights support the notion that, in high-risk or post-resection CRC populations, dietary interventions or microbiota-directed formulations that enrich beneficial AhR ligands could provide a low-toxicity, long-term strategy to “ecologically” tune ILC3 toward barrier-protective profiles. Retinoic acid itself has been shown to directly reshape ILC plasticity in humans. In relapsing-remitting multiple sclerosis, ex vivo stimulation of peripheral “helper” ILC with IL-2, IL-33, and retinoic acid reprograms the ILC spectrum, increasing ILC1 frequency and IL-10 production while decreasing ILC2 and CCR6^+^ subsets, with these changes correlating with disease activity indices (98). Although the disease context differs from CRC, this work underscores that retinoic acid can reconfigure not only ILC residency and proliferation but also the functional distribution of ILC subsets. Translating these concepts to CRC, one can envision combined regimens in which IL-12/IL-23 inhibitors provide a “hard” brake on inflammatory skewing, while retinoic acid and beneficial AhR ligands offer a “soft” ecological push toward IL-22-dominant ILC3 states.

### 6.3 Reinforcing barrier function

**Figure 2:**
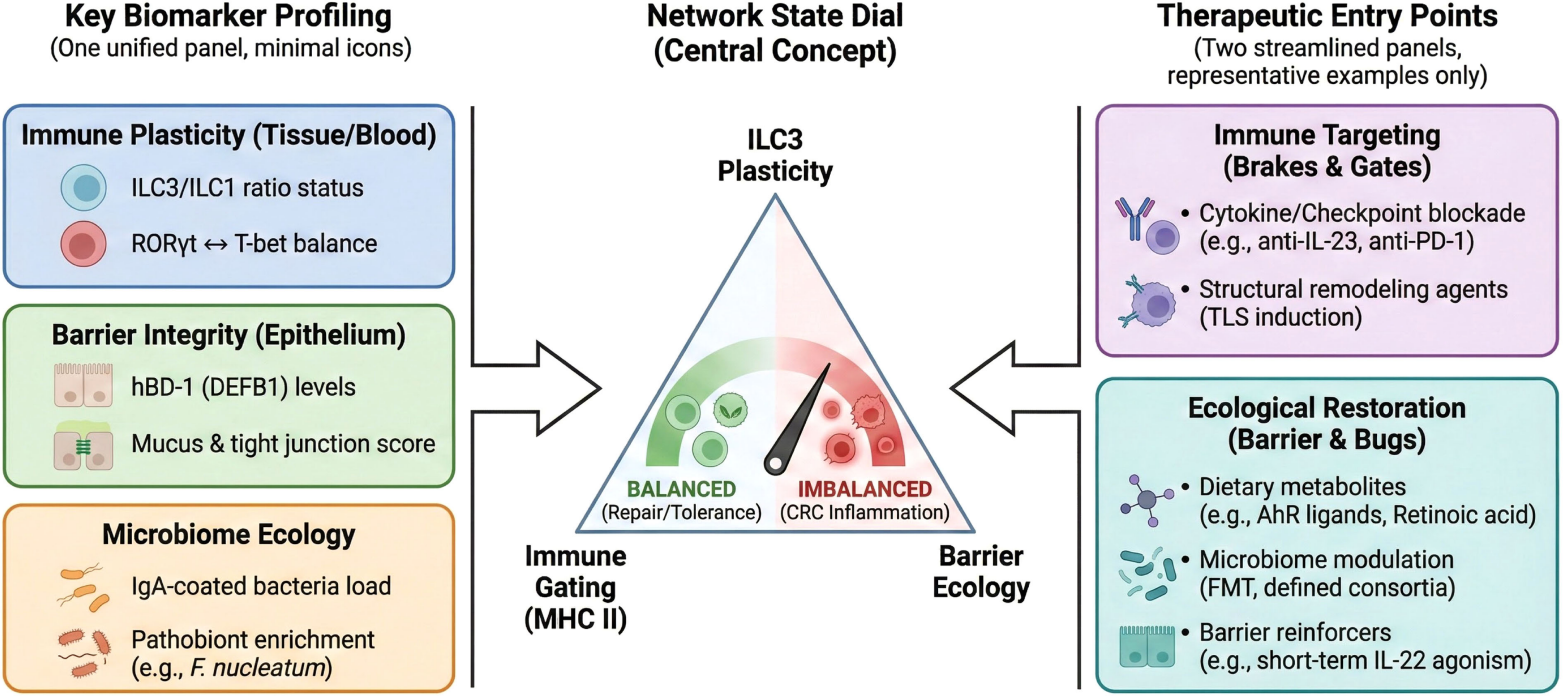
Biomarker layers and therapeutic entry points to reset the ILC3–barrier–microbiota network. Multi-compartment readouts are organized across tissue, blood, and epithelial–microbiome profiles. Therapeutic nodes are mapped to network control points, including IL-23 pathway attenuation, OSM/OSMR interruption, checkpoint modulation, ecological tuning, microbiome-precision strategies, and time-limited barrier reinforcement, with TLS induction as a structural amplifier of adaptive immunity.

Compared with plasticity, ILC3 antigen presentation–like activity and their coupling to epithelial barrier function may seem less amenable to direct pharmacological targeting. Nonetheless, several experimental therapies and barrier-focused strategies begin to sketch a pharmacological blueprint for this axis. On the IL-22 side, efmarodocokin alfa, an IL-22–Fc fusion protein, has shown in phase I/II trials in healthy volunteers and patients with ulcerative colitis that repeated intravenous administration can safely induce a broad set of epithelial IL-22 target genes, including antimicrobial peptides, mucins, and tight junction molecules, without causing systemic immunosuppression or serious adverse events (99). Although not yet tested in CRC, its profile in inflamed mucosa suggests that short-term use of IL-22 analogues in high-risk or post-surgical patients could be feasible as a barrier-repair measure. Upstream regulators such as HIF-1α stabilizers can indirectly influence IL-22 and antimicrobial outputs through oxygen-sensing transcriptional networks. Pharmacological inhibition of epithelial prolyl hydroxylases to stabilize HIF-1α promotes mucosal healing by increasing integrin α6 and α2 expression, enhancing epithelial migration and proliferation, and accelerating recovery from DSS-induced injury (100). In combination with an intact ILC3–IL-22 axis, such agents could provide a synergistic “window” in which epithelial repair, antimicrobial peptide upregulation, and microbiota rebalancing are facilitated, creating more favorable conditions for restoration of ILC3–MHCII function. Barrier “hardware” can also be upgraded via metabolic and antimicrobial peptide–oriented interventions. Targeting polyamine metabolism has revealed that mice lacking spermine oxidase develop more severe colitis and higher tumor burden in DSS and AOM–DSS models, accompanied by excessive α-defensin expression and dysbiosis; oral spermidine supplementation reverses these phenotypes, reducing inflammation and tumor incidence and partially restoring microbial balance (101). Although focused on α-defensins, this work illustrates how metabolic tuning of defensin profiles and microbiota can reset the equilibrium among immunity, barrier, and microbial communities. Natural products offer more direct antimicrobial peptide modulation. In a model of cyclophosphamide-induced immunosuppression and intestinal barrier damage, oral administration of Alhagi honey polysaccharides improved spleen and thymus indices and Peyer’s patch numbers, increased IL-2, IL-6, and TNF-α, upregulated intestinal β-defensin expression, restored tight junction and mucin levels, normalized villus height and crypt depth ratios, and increased short-chain fatty acid concentrations (102). While far from immediate CRC application, this “natural product–defensin–barrier” pattern suggests a design principle: future small molecules, peptides, or polysaccharide-based agents that selectively induce hBD-1 and related antimicrobial pathways, combined with HIF-1 stabilizers and IL-22 analogues, could transform the ILC3–MHCII–barrier axis from a passive repair system into an actively reinforced defensive front (**Figure 2**).

### 6.4 Combination logic

The practical challenge is how to integrate these concepts with existing immunotherapy and chemotherapy. Recent reviews of checkpoint-based combinations in CRC emphasize that, outside MSI-high disease, most tumors respond poorly to PD-1/PD-L1 monotherapy and that microbiota composition, myeloid polarization, and innate immune pathway abnormalities are key determinants of resistance and heterogeneity (103). Accordingly, future regimens are expected to move toward “PD-1/PD-L1 blockade plus microenvironmental reprogramming,” encompassing microbiota modulation, metabolic interventions, and targeting of innate immune cells. Complementary microbiome-focused overviews argue that multi-omics monitoring, AI-assisted analysis, fecal microbiota transplantation, probiotics, and metabolite-targeted strategies will underpin “microbiome precision medicine” aimed at enhancing immunotherapy responsiveness and reducing toxicity in gastrointestinal cancers (104). In CRC specifically, reintroducing the “ILC3–IL-22–barrier” component has begun to show preclinical promise. In one study, CRC expressed high levels of C5aR1, which correlated with poor prognosis. Pharmacological C5aR1 blockade repolarized tumor-associated macrophages toward an M1-like state via the AKT2/PFKLM axis. This blockade also increased serum IL-22 levels, elevated antimicrobial peptide mRNA in the gut, and reshaped microbiota composition in a TLR5-dependent manner, altogether enhancing antitumor immunity (105). This chain—from complement inhibition to IL-22 and antimicrobial peptide upregulation, then to microbiota and myeloid reprogramming—provides a concrete example of integrating the ILC3 axis into broader microenvironmental modulation.

At the interface of cell therapy and chemotherapy, ongoing strategies seek to recruit innate lymphocytes. A randomized trial combining IL-2–activated NK cell infusions with adjuvant XELOX chemotherapy in stage III CRC aims to improve progression-free and overall survival by augmenting innate cytotoxic capacity and reducing recurrence and metastasis (106). In parallel, “memory-like” NK cells generated by ex vivo priming with IL-12, IL-15, and IL-18 show superior IFN-γ production and cytotoxicity against CRC targets in both autologous and allogeneic systems compared with conventional NK cells, and have been proposed for clinical testing in metastatic CRC unresponsive to checkpoint inhibitors (107). Although these approaches currently focus on NK cells, closely related innate lymphoid populations such as ILC3 could be considered in future “memory-like” or adoptive transfer designs. More directly aligned with the present review is the proposal to treat ILC3 as next-generation immunotherapy targets. Given their high plasticity and multi-lineage functions, ILC may act either as powerful allies of T cells in antitumor responses or as tools to restore gut homeostasis and mitigate immune-related toxicity, depending on how precisely their lineage transitions and cytokine outputs are controlled (108). Synthetic-biology–inspired induction of mature tertiary lymphoid structures (mTLS) in CRC—via local chemokine delivery or engineered scaffolds—has been shown to increase mTLS abundance, enhance T and B cell infiltration, and improve PD-1 inhibitor responses and survival (109). Considering the association of NKp44^+^ ILC3 with tertiary lymphoid structures, combining mTLS-inducing strategies with preservation or restoration of local ILC3 function offers a blueprint for “structural” immune remodeling rather than simply dose escalation of systemic drugs.

Overall, integrating ILC3 plasticity, MHCII-dependent antigen presentation, and the epithelium–microbiota–hBD-1 axis suggests a potential combination path: In phases of controllable tumor burden, a sequential strategy can gently pull ILC3 back toward IL-22-dominant states. To achieve this, clinicians could use IL-23, retinoic acid, AhR agonists, and metabolic–nutritional strategies. Concurrently, they can strengthen the epithelial barrier and broaden the antimicrobial spectrum with IL-22 analogues, HIF-1 stabilizers, and defensin modulators. Subsequently, they can overlay additional therapies, including PD-1/PD-L1 inhibitors, memory-like NK or other innate lymphocyte therapies, microbiome-precise interventions, and TLS induction. Such a program will require stepwise development, but it offers a coherent temporal and spatial framework for the central idea of “resetting ILC3, stabilizing the barrier, and correcting the microbiota” in CRC.

## 7 Conclusion

Taken together, the immunological landscape of colorectal cancer (CRC) can be viewed as a highly coupled tri-directional network rather than a collection of isolated pathways. One axis centers on ILC3 plasticity, linking IL-22, IL-17, and interferon-γ outputs to epithelial repair, microbial control, and T cell polarization. A second axis is defined by MHCII-dependent, antigen presentation–like functions of ILC3, which translate recognition of commensals and carcinogenic microbes into shifts in Th17/Treg balance and IgA specificity. The third axis is the epithelium–microbiota–hBD-1 barrier system, which continuously sculpts microbiota composition and mucosal inflammatory thresholds at the level of both physical and chemical barriers. In this framework, CRC emerges as a syndrome of multiple interlocking loops drifting out of balance: an IL-22/OSM/STAT3 circuit that shifts from acute protection to chronic pro-tumor signaling; ILC3-to-ILC1 reprogramming that trades barrier-supportive IL-22 for tissue-damaging IFN-γ; weakening of ILC3–MHCII–driven antigen focusing for T cells and IgA; and downregulation or functional saturation of hBD-1/DEFB1, which lowers antimicrobial selection pressure at the mucosal surface. Each disturbance may appear modest in isolation, but together they reinforce one another and stabilize a barrier–microbiota–immune state that favors tumor persistence. Under this network-based view, asking whether ILC3 or hBD-1 are “good” or “bad” in CRC is no longer meaningful. Their roles depend on stage, anatomical location, microbial context, and treatment exposure. The key determinants are their relative weights within the network: whether ILC3 are maintained in an IL-22-dominant, metabolically balanced state; whether MHCII-dependent antigen presentation remains sufficiently focused on relevant microbial and tumor antigens; whether hBD-1 still provides a basal antimicrobial “background”; and whether the microbiota can still be shaped by IgA and defensins rather than driving inflammation and carcinogenesis. This also frames the central unresolved questions. For ILC3–MHCII, human data are still insufficient to define how essential these cells are as APC in tumors, and how they interact with other RORγt^+^ MHCII^+^ populations in different regions of the tumor–draining lymphoid axis. For ILC3-to-ILC1 plasticity, the net effect may change over time, as IFN-γ–dependent cytotoxicity is weighed against epithelial damage, myeloid suppression, and checkpoint upregulation, with PD-1 and CTLA-4 acting as metabolic and transcriptional “safety valves” on ILC as well as on T cells. For DEFB1/hBD-1, *in vitro* data support a tumor-suppressive role via mTOR, autophagy, and cell death pathways, whereas patient cohorts suggest that high DEFB1 expression can also mark barrier stress and checkpoint-dominated microenvironments. Addressing these issues will require longitudinal sampling across normal mucosa, adenomas, early cancers, and advanced disease, with DEFB1, IL-22/OSM/STAT3 activity, microbiota composition, and IgA targeting mapped in parallel.

From a translational standpoint, a coherent theme emerges: “pulling back” ILC3, stabilizing the barrier, and correcting the microbiota. Pulling back ILC3 does not simply mean increasing their numbers, but reshaping their niche so that IL-12/IL-23, retinoic acid, AhR ligands, and checkpoint signals keep them in an IL-22-dominant, MHCII-competent, non-exhausted state rather than pushing them into IFN-γ-high or functionally silent extremes. Stabilizing the barrier involves short-window use of IL-22-based agents, HIF-1α stabilizers, and modulators of hBD-1 and other antimicrobial peptides to prevent bacterial translocation and inflammatory escalation, particularly in preneoplastic lesions, perioperative mucosal repair, or treatment-induced damage, rather than in late-stage tumors where IL-22/STAT3 signaling is already co-opted by cancer cells. Correcting the microbiota requires moving beyond generic “probiotics” toward individualized reshaping based on multi-omics, IgA-coating patterns, and defensin profiles, so that microbial communities shift from co-drivers of carcinogenesis back toward partners in barrier maintenance and immune homeostasis.

In practice, the most realistic future path is unlikely to be a single “magic bullet” but a temporally structured combination strategy. At diagnosis, biomarker panels capturing ILC3 plasticity, MHCII function, and barrier status could define baseline network states. During perioperative and adjuvant phases, IL-23/retinoic acid/AhR modulation and nutritional or microbiome-directed interventions could gently re-equilibrate ILC3 and microbial communities. In systemic therapy, PD-1/PD-L1 blockade, innate lymphocyte–based approaches, induction of tertiary lymphoid structures, and barrier-strengthening interventions may then be layered in a staged or adaptive fashion to enhance antitumor immunity while avoiding irreversible mucosal toxicity. In this sense, the “ILC3 plasticity–antigen presentation–epithelium/microbiota/hBD-1” model is not only a conceptual map of CRC immunobiology but also a practical wiring diagram for future experiments and trials. The main challenge is less about discovering new molecules than about learning how to manipulate this existing network with enough precision and timing to alter its trajectory without triggering new, hard-to-control modes of imbalance.
