# Correction: Plasticity and antigen presentation by group 3 innate lymphoid cells in colorectal cancer

**DOI:** 10.3389/fimmu.2026.1922676

**Published:** 2026-07-21

**Authors:** Lu Qiao, Jingwen Zhang, Jiaxi Li, Xia Li, Xiaoxia He, Mingyu Zhang, Jingkun Lu, Xuan Zhang, Jing Dong, Gesi Tao, Yue Wang, Jiaxian Cui, Lili Bao, Pengwei Zhao

**Affiliations:** 1 Laboratory of Microbiology and Immunology, School of Basic Medical Science, Inner Mongolia Medical University, Hohhot, China; 2 Department of Clinical Laboratory, Affiliated Hospital of Inner Mongolia Medical University, Hohhot, China

**Keywords:** antigen presentation, colorectal cancer, group 3 innate lymphoid cells, human beta-defensin 1, microbiota

In “Lili Bao and Pengwei Zhao” the affiliation was wrong.

Affiliation “1Laboratory of Microbiology and Immunology, School of Basic Medical Science, Inner Mongolia Medical University, Hohhot, China” was erroneously given as “2Department of Clinical Laboratory, Affiliated Hospital of Inner Mongolia Medical University, Hohhot, China”.

The original version of this article has been updated.

